# Detection and Tracking of SARS-CoV-2 Lineages through National Wastewater Surveillance System Pathogen Genomics

**DOI:** 10.3201/eid3113.241411

**Published:** 2025-05

**Authors:** Dorian J. Feistel, Rory Welsh, Jeffrey Mercante, Miguella Mark-Carew, Jason Caravas, Arun Boddapati, Samantha Sevilla, Matthew H. Seabolt, Dhwani Batra, Suchitra Chavan, Shatavia Morrison, Jesse Yoder, Hannah Long, Satvik Mishra, Benjamin Lorentz, Andi Dhroso, Iryna V. Goraichuk, Seonghye Jeon, Daniel M. Cornforth

**Affiliations:** Author affiliations: Centers for Disease Control and Prevention, Atlanta, Georgia, USA (D.J. Feistel, R. Welsh, J. Mercante, M. Mark-Carew, J. Caravas, M.H. Seabolt, D. Batra, S. Morrison, S. Jeon, D.M. Cornforth); Leidos Inc., Reston, Virginia, USA (A. Boddapati, S. Sevilla, S. Chavan); DCI Solutions, Aberdeen Proving Ground, Maryland, USA (J. Yoder); Palantir Technologies Inc., Denver, Colorado, USA (J. Yoder, H. Long, S. Mishra); Goldbelt Professional Services, Chesapeake, Virginia (B. Lorentz); ASRT Inc., Smyrna, Georgia, USA (A. Dhroso, I.V. Goraichuk)

**Keywords:** wastewater surveillance, computational biology, SARS-CoV-2, COVID-19, severe acute respiratory syndrome coronavirus 2, viruses, respiratory infections, wastewater-based epidemiological monitoring, public health surveillance, bioinformatics, zoonoses

## Abstract

We conducted retrospective analysis of the emergence of the SARS-CoV-2 JN.1 variant in US wastewater during November 2023–July 2024 using Aquascope, a bioinformatics pipeline for the National Wastewater Surveillance System. This study highlights the value of open-source bioinformatics tools in tracking pathogen variants for public health monitoring.

The emergence and rapid global spread of SARS-CoV-2 emphasized the need for efficient methods of identifying and tracking viral changes as they circulate within communities. Wastewater pathogen genomic surveillance offers a timely, noninvasive, and cost-effective method for detecting pathogen genetic material in sewersheds, providing a comprehensive snapshot of community transmission dynamics to monitor infection trends (*1*). Wastewater surveillance complements clinical surveillance and can identify viruses shed by persons who are presymptomatic, asymptomatic, or not tested in healthcare facilities, making it a robust measure of overall prevalence of SARS-CoV-2 lineages in circulation, the early geographic spread of emerging variants already detected in humans, and novel variants of SARS-CoV-2 not yet detected in humans (*2*).

In 2020, the Centers for Disease Control and Prevention (CDC) established the National Wastewater Surveillance System (NWSS) to track the spread of SARS-CoV-2 in wastewater at the local level (*3*). Since then, laboratories in academia and public health have made considerable advancements in tools for characterizing pathogen genomic variation in wastewater (*3*–*6*). Through wastewater sequencing, NWSS monitors genetic variation in SARS-CoV-2, identifying variants and mutations that may affect disease severity or efficacy of PCR-based diagnostics, vaccines, or therapeutics (*3*). To enable timely, reproducible, and high-throughput analyses of wastewater sequence data for SARS-CoV-2 monitoring, NWSS collaborated with CDC’s Scientific Computing and Bioinformatics Services in the Division of Infectious Disease Readiness and Innovation, National Center for Emerging and Zoonotic Infectious Diseases, to develop the bioinformatics pipeline Aquascope, modeled after the CFSAN Wastewater Analysis Pipeline (C-WAP) (*5*). Aquascope will replace C-WAP on the CDC 1CDP platform, providing timely results to jurisdictions and the public. Aquascope is more robust than C-WAP; it includes quality metrics and logging features and can be deployed in high-performance computing and cloud platforms. Implemented in Nextflow, Aquascope uses open-source, containerized bioinformatic tools for quality control, variant identification, and estimation of lineage abundance from tiled-amplicon short-read and long-read wastewater sequence data. 

## Case Study

As a case study, we sought to retrospectively track the spread of the JN.1 variant of SARS-CoV-2, a closely related descendant of BA.2.86, first detected in clinical samples in early September 2023 (*2*). By early December 2023, JN.1 had become the predominant variant in the United States. Using Aquascope, we estimated the relative abundance of known SARS-CoV-2 lineages from wastewater sequence data collected from across the country. This activity has been reviewed by CDC and determined to be nonresearch public health surveillance that did not require review through the CDC Human Research Protection Office or Institutional Review Board.

We used a subset of wastewater surveillance data collected by Verily Life Sciences (https://verily.com) on behalf of NWSS (National Center for Biotechnology Information BioProject no. PRJNA1027353) to estimate variant relative abundances. By limiting analysis to data from this BioProject, we ensured consistency in laboratory methods across the data analyzed. We included in our analysis collection weeks with >10 samples, comprising 3,377 unique samples gathered from 130 sites across 87 counties in 32 US jurisdictions. The collection period was November 13, 2023–July 23, 2024. All samples were concentrated, DNAse treated, and reverse transcribed before amplification using the NEB Q5 High-Fidelity PCR kit with ARTIC version 5.3.2 primers (New England Biolabs, https://www.neb.com). Sequencing libraries were prepared with the NEBNext Ultra II DNA Library Prep Kit and pair-end sequenced (2 × 300 bp) on the Illumina NextSeq 2000 (Illumina, https://www.illumina.com).

We processed raw sequencing data using Aquascope version 2.1.0, first performing quality checks, then removing adapters and low-quality regions. We aligned reads to the SARS-CoV-2 reference genome (GenBank accession no. MN908947.3) and trimmed primers used for amplification. We estimated the relative abundance of known SARS-CoV-2 lineages using Freyja (*6*) with SARS-CoV-2 UShER barcodes from July 26, 2024 (*7*). Full pipeline details are publicly available (https://github.com/CDCgov/aquascope). Lineage relative abundance estimates correspond to samples collected across jurisdictions within the same week after lineage aggregation and normalization; lineages representing <5% of the total being aggregated were categorized as Other. We tracked all lineage abundances, and we aggregated sublineages not enumerated with their parent lineages on the basis of Pango lineage definitions (*8*). We chose parent lineages to reflect those displayed on the CDC COVID Data Tracker (*9*) and NWSS dashboard (*10*). 

Our analysis of wastewater sequence data revealed a distinct temporal trend in the emergence and spread of the JN.1 variant (Figure). JN.1 was first detected by the pipeline in a sample collected on November 15, 2023; however, because this week had <10 samples collected, the earliest displayed data are from samples collected the subsequent week. After initial detection, JN.1 increased in prevalence in early December 2023, peaked in early March 2024, and continued to decline through late July in the final displayed weeks. Results also showed other known lineages, such as the JN.1 sublineages JN.1.7 and JN.1.11.1, emerging sequentially and maintaining a significant presence. KP.2 and KP.3 lineages also appeared and grew to varying levels of prevalence. Our study of the JN.1 lineage with Aquascope demonstrates the ability of CDC’s NWSS to monitor emerging SARS-CoV-2 variants.

**Figure:**
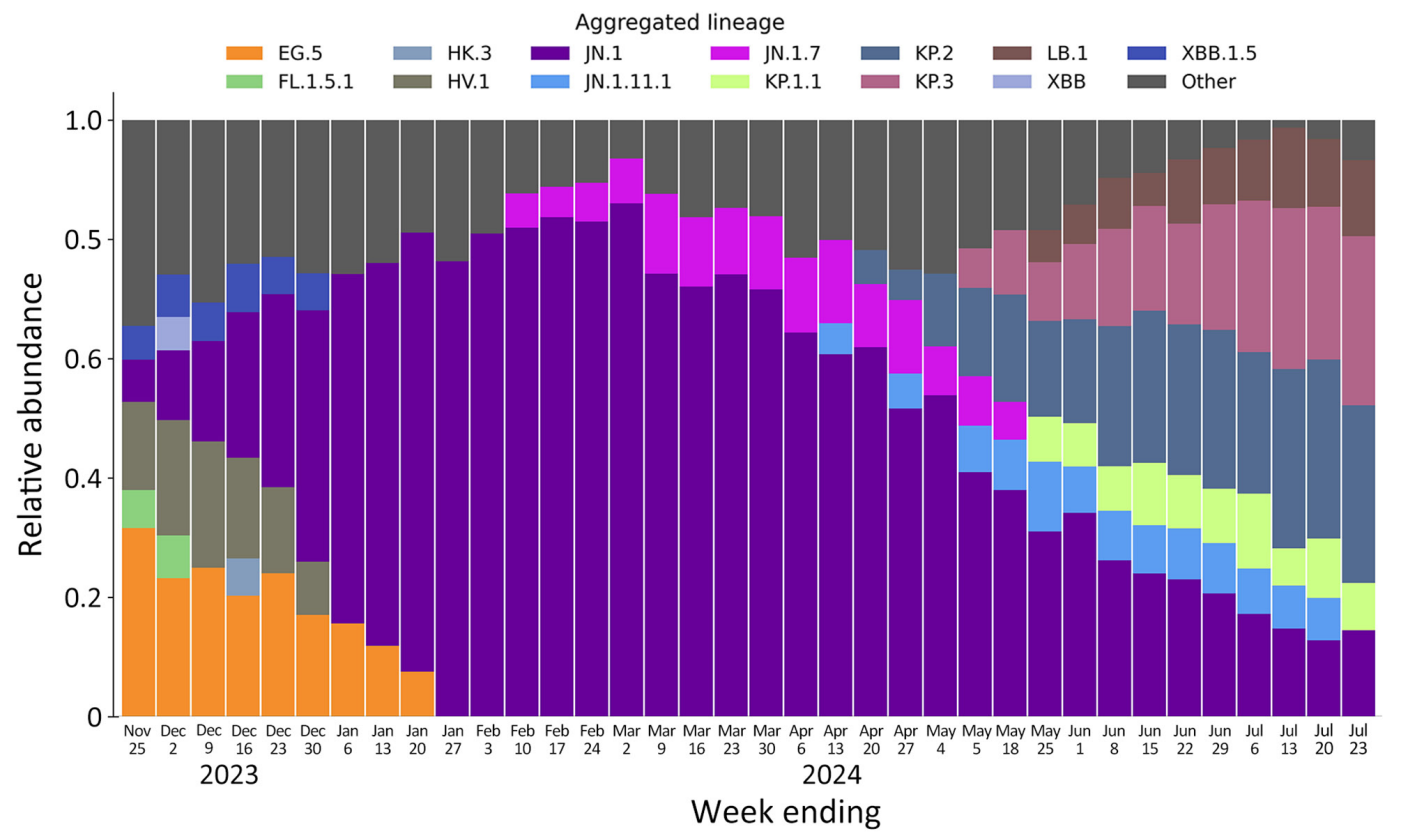
Average relative abundance of aggregated SARS-CoV-2 lineages detected in wastewater samples collected across the United States for the weeks ending November 25, 2023–July 23, 2024. The final time point shown (July 23, 2024) does not represent a full week of data.

## Conclusions

Advances in wastewater bioinformatics pipelines, such as Aquascope, enhance our ability to track public health outbreaks by providing a relatively passive, low-cost, near–real-time surveillance approach that complements clinical genomic surveillance. Although JN.1 was first detected in a US clinical sample collected in late September 2023 (*11*), earlier than the first samples identified with Aquascope here, we note that the Bioproject analyzed in this study does not contain data preceding November 13, 2023. Still, trends in JN.1 proportions inferred in NWSS samples by Aquascope were similar to those in clinical sequence data, which first surpassed 0.1% prevalence in November 2023 and surpassed 50% prevalence from early January to the end of April 2024, similar to wastewater trends (Figure) (*11*).

Future work will cross-compare wastewater and clinical data using additional NWSS sites and account for differences in coverage, population normalization, and other analytical considerations. Although the pipeline we describe focuses on SARS-CoV-2 lineage abundance, Freyja’s deconvolution algorithm (*6*) can use barcode libraries from additional pathogens to estimate their abundances in mixed wastewater samples, so that Aquascope can be adapted for broader pathogen detection. This pipeline relies on prior characterization of SARS-CoV-2 lineages; future advancements may enable identification of previously uncharacterized lineages. 

One potential challenge for personal use of Aquascope is computational requirements robust enough to support large input datasets; it requires a high-performance computing environment with support for required dependencies. Aquascope will soon operate within a larger, scalable CDC computing platform freely available to public health partners for wastewater surveillance efforts. Continuous development of bioinformatics pipelines like Aquascope will broaden our capacity to monitor emerging infectious disease threats through wastewater surveillance.
